# The Antibacterial Effect of Selected Essential Oils and Their Bioactive Constituents on *Pseudomonas savastanoi* pv. *savastanoi*: Phytotoxic Properties and Potential for Future Olive Disease Control

**DOI:** 10.3390/microorganisms11112735

**Published:** 2023-11-09

**Authors:** Laura Košćak, Janja Lamovšek, Edyta Đermić, Sara Godena

**Affiliations:** 1 Laboratory for Plant Protection, Department of Agriculture and Nutrition, Institute of Agriculture and Tourism, Carlo Hugues 8, 52440 Poreč, Croatia; laura@iptpo.hr; 2 Agricultural Institute of Slovenia, Hacquetova ulica 17, 1000 Ljubljana, Slovenia; janja.lamovsek@kis.si; 3 Department of Plant Pathology, Division of Phytomedicine, Faculty of Agriculture, University of Zagreb, Svetošimunska cesta 25, 10000 Zagreb, Croatia; edermic@agr.hr

**Keywords:** biopesticides, carvacrol, *Olea europaea* L., olive knot disease, phytotoxicity, plant disease management, plant metabolites

## Abstract

Plant pathogenic bacteria pose a significant threat to olive cultivation, leading to substantial economic losses and reduced yield. The efficacy of antimicrobial agents against these pathogens is of great interest for sustainable disease management strategies. As such, the management of olive knot disease is one of the major challenges in olive protection. In the presented study, through a series of in vitro assays, we investigated the antimicrobial effect of six essential oils (EOs) and their most concentrated constituents against causative agent of olive knot disease—*Pseudomonas savastanoi* pv. *savastanoi*, highlighting the high potential of *Origanum compactum* EO and its constituent carvacrol. Carvacrol exhibited the highest potential for practical application, demonstrating membrane disruption as its mechanism of action even at the lowest concentration. The bactericidal effect of antimicrobials was confirmed in a time–kill assay, where concentrations of MIC, 2× MIC, and 4× MIC were evaluated. Some of the applied treatments resulted in inhibition equal or higher than copper-based treatment. Additionally, we assessed the phytotoxicity of carvacrol by foliar application on olive cv. Leccino. The appearance of phytotoxic injuries majorly occurred on the young leaves of olive plants, with the highest proportion of damaged canopy observed when the 2× MIC concentration was applied. Due to its great efficiency against *P. savastanoi* pv. *savastanoi* in vitro, these findings highlight the potential of carvacrol as a molecule of interest for the development of environmentally friendly biopesticides. This study also contributes to the advancement of disease management practices in olive cultivation, leading to enhanced crop protection.

## 1. Introduction

The use of conventional pesticides in agriculture has raised concerns regarding their adverse effects on humans, animals, and the environment. However, meeting the increasing demands for high crop yields, while ensuring sustainability, poses significant challenges in agriculture [1]. As such, olive knot disease, which is a widespread disease that affects olive orchards, is caused by Gram-negative bacterium *Pseudomonas savastanoi* pv. *savastanoi* (*Pss*), and it is one of the most challenging olive diseases to control [2,3]. Despite being a persistent pathogen in olive orchards for centuries, there is currently no effective control management strategy to eradicate this bacterium. The control of phytopathogenic bacteria primarily relies on the use of copper-based preparations, as the use of antibiotics in agriculture is generally prohibited in many agricultural areas. This situation is worsened by the lack of selective bactericidal preparations. Some available microbial-based preparations are present on the market, but their efficacy may not always meet the desired levels of disease suppression in commercial production [4]. Therefore, there is an urgent need to develop effective and sustainable formulations to control olive bacterial diseases and mitigate the impact of olive knot disease. Still, the standard copper-based preparations can be effective when used correctly; improper use can lead to phytotoxicity and long-term environmental contamination. Moreover, the emergence of bacterial resistance to various antimicrobials through horizontal gene transfer or mutations is an additional growing concern [5,6].

Faced with the mentioned problems in agriculture, scientists have prompted the exploration of alternative solutions for plant protection strategies. Essential oils (EOs) and the constituents present in their chemical profiles have emerged as promising substitutes for conventional pesticides due to their well-established antimicrobial properties [7]. EOs are secondary plant metabolites with complex mixtures of terpenoids, alcohols, phenols, aldehydes, ketones, esters, acids, and many other bioactive organic constituents. Specifically, terpenoids have been recognized as interesting molecules for their antimicrobial activity, making EOs suitable candidates for bacterial control [7,8]. However, the composition of EOs varies depending on the growth conditions of plants, including the climate, plant genetics, cultivation practices, and physiological condition of plants [9]. The EOs derived from plants belonging to the Lamiaceae family, e.g., genera *Mentha*, *Melissa*, *Salvia*, *Thymus*, and *Origanum*, showed effective control of numerous economically important phytopathogenic bacteria [9,10,11,12,13,14,15,16,17,18,19,20]. Generally, EOs derived from different *Mentha* and *Salvia* taxa exhibit lower antibacterial activity. Interestingly, antibacterial effect is dependent on plant variety, and thus EOs that are obtained from different plant varieties can exhibit equal or even stronger antibacterial effect than antibiotics, even though the majority of other cultivars from the same genera show less potential as antimicrobials. The greater antibacterial efficiency of *Mentha* EOs has been found to be significantly dependent on high concentrations of menthol or menthone in the chemical profile of this plant variety [7,9]. Similarly, the antibacterial properties of the *Thymus* and *Origanum* species are mostly attributed to the presence of the isomeric phenols thymol and carvacrol [21]. Furthermore, the antimicrobial activity of various compounds is generally more effective against Gram-positive bacteria, presumably because the presence of an additional outer membrane in cell wall structure of Gram-negative bacteria that can restrict the entry of antimicrobial compounds [7,22,23]. However, the inhibitory effects of EOs can significantly vary depending on the bacterial species and even the pathovar within the species, regardless of the complexity of their cell structures [9,10,13], as well as method used for this purpose [24].

Given these challenges, there is an urgent need to evaluate and confirm the potential of plant metabolites as alternatives to current plant protection agents, including copper-mediated protection against *Pss*. Our research aims to address this need by investigating the in vitro antimicrobial effect of selected EOs and the most concentrated constituents of EOs on *Pss* through a series of in vitro experiments. Our aim was to determine the minimal inhibitory concentration (MIC) and minimal bactericidal concentration (MBC) of the tested antimicrobials and compare bacterial susceptibility to conventional antibiotics and copper-based commercial agents. In this study, we investigate whether the antimicrobials are bacteriostatic or bactericidal agents and confirm their mechanisms of action by evaluating the time of death by time–kill assay and the effect on cellular metabolites. In the greenhouse experiment we aimed to determine the phytotoxic properties of carvacrol on 2–3-year-old cv. Leccino olive plants. To the best of our knowledge, this is the first experiment of its kind conducted on olive plants specifically focusing on the determination of phytotoxicity and monitoring *Pss* growth and death through time and a series of concentrations of tested agents. These experiments contribute to the understanding of the efficacy and safety of these agents for potential use in the control of olive knot disease.

## 2. Materials and Methods

### 2.1. Preparation of Bacterial Inoculum

The reference strain of the bacterium *P. savastanoi* pv. *savastanoi*—CFBP5075 (Italy) was purchased from the National Institute of Agricultural Research, INRA (Paris, France). To maintain the bacterial cultures, stock cultures were prepared by sub-culturing on solid King’s B (KB) growth medium (King Medium B, Pseudomonas Agar F, Biolife, Milano, Italy). The plates were incubated at 27 °C for 24–48 h. Pure colonies were selected and transferred to liquid KB medium [25] and the bacterial suspensions were incubated overnight in a shaker-incubator set to 27 °C and 80 rpm.

### 2.2. Qualitative Determination of Antibacterial Effect

Antibacterial susceptibility was tested using the disc diffusion method, following the Kirby–Bauer protocol [26]. Solid KB growth medium was inoculated with 100 µL of bacterial suspension with bacterial density adjusted to 10^8^ CFU/mL using the McFarland Equivalence Turbidity Standard 0.5 (Remel, UK). Sterile antibiotic discs (6 mm in diameter; Whatman AA Discs, PLC, Cytiva, China) were aseptically placed on the plates, and aliquots of 15 µL of pure or diluted antimicrobials chosen for testing in sterile distilled water were pipetted onto the discs under a laminar flow (NUVE LN 090, Ankara, Turkey). Sterile distilled water (SDW) was used as a negative control. Two reference treatments, i.e., antibiotic tetracycline (Fisher Scientific, Hampton, NH, USA) and a copper-based preparation of Nordox 75 WG (Syngenta, Basel, Switzerland) were included for comparison with the tested compounds. Prior to the application of the treatments on the discs, the suspensions were thoroughly vortexed and pipetted. The diffusion of antimicrobials was assessed by incubating the plates at +4 °C for two hours. After diffusion, the plates were incubated overnight at 27 °C in the dark. After 24 h of incubation, the diameter of the inhibition zones was measured in millimeters using a caliper.

The antimicrobials included in the study involved the use of six commercially available EOs (Pranarōm, Ath, Belgium) and their chemical constituents (carvacrol, linalyl acetate, DL-menthol, and (-)-terpinen-4-ol (Thermo Fisher Scientific Inc., Waltham, MA, USA), thymol (VWR International, Leuven, Belgium), and α,β-thujone (Sigma Aldrich, Burlington, MA, USA); see Table S2 for details). These treatments were as follows: C1—pure/undiluted liquid of EOs or constituents or diluted solid form of the constituents; C2—diluted form of the EO or EO constituent (as specified in Table S1)—mg/mL; and C3—diluted form of the EO or EO constituent at a concentration of 5% (*v*/*v*).

### 2.3. Quantitative Determination of Antibacterial Effect

The minimal inhibitory concentrations (MIC) of antimicrobials were determined using the broth dilution method. Different concentrations of the treatments were prepared in 2 mL of liquid KB medium and mixed with 2 mL of an overnight bacterial culture. The bacterial density was adjusted to approximately 10^6^ CFU/mL (optical density (OD_600_) 0.15) [27]. The MICs were determined by visual assessment of the tube turbidity, which was interpreted as no visible growth after 24 h incubation period. The minimal bactericidal concentration (MBC) was defined as the concentration at which a 99.9% reduction in bacterial growth was determined after plating treatments on solid agar plates (KB medium) and observing growths after 24 h of incubation. To further characterize the antimicrobial effect, the bacteriostatic and bactericidal nature of the antimicrobials was assessed by calculating the ratio of MBC to MIC. A quotient greater than four indicated bacteriostatic properties, while a value equal or lower value indicated a bactericidal action [28].

To compare the antibacterial potential of the tested antimicrobials with antibiotic and copper-based preparations, the percentage of bacterial growth inhibition at the MIC values was calculated using the spectrophotometric values of absorbance of treatments at 600 nm wavelength (OD). The inhibition was calculated by using the following formula:

Inhibition (%) = [(A_600_ − B_600_)/A_600_] × 100 (1)

where A_600_ represents the absorbance of the control (bacterial culture without antimicrobial treatment) and B_600_ represents the absorbance of the culture with tested antimicrobial at a specific dilution. The liquid KB medium served as a blank. The concentrations used for the potential antimicrobials were prepared through two-fold serial dilutions ranging from 20 to 0.3125 mg/mL for *Thymus vulgaris* and *Origanum compactum* EOs and 40 to 0.625 mg/mL for *Mentha* × *piperita*, *Origanum majorana*, *Salvia officinalis*, and *Salvia sclarea* EOs, whilst the concentrations of constituents ranged from 5.0 to 0.078125 mg/mL.

### 2.4. Time–Kill Assay

To determine the time at which bacterial cells die after treatment exposure we performed the time–kill assay, which was conducted following the guidelines provided by the Clinical and Laboratory Standards Institute [29] for testing antimicrobial activity, with a few modifications. The determined MIC of the antimicrobials, as well as concentrations two and four times higher than the MIC, were used for the experiment. For the test, 100 µL aliquots of the MIC concentration diluted in sterile saline (1:9 *v*/*v*) and aliquots of 10 µL were plated onto solid KB growth medium at various time points after exposure to treatment (0, 1, 2, 4, 6, and 24 h). After 24 h we counted the CFU and determined the bacterial concentration in CFU per milliliter. The CFU/mL values were transformed to a logarithmic scale to create time–kill plots. A decrease in CFU/mL by ≥3log over time was considered as bactericidal action of treatment.

### 2.5. Leakage of Cellular Metabolites

To evaluate the leakage of bacterial cellular metabolites as a possible mode of action, we followed the methodology outlined by Miksusanti et al. [30] and Elcocks et al. [31] with some modifications. Initially, a bacterial culture (10^6^ CFU/mL in 10 mL of liquid KB medium) was prepared and transferred into centrifuge tubes. The tubes were then centrifuged three times in a row at 4800 rpm for 15 min. After each centrifugation step, the supernatant was carefully discarded and medium was added to the bacterial pellet. Following the centrifugation process, a total volume of 3 mL of the obtained bacterial suspensions was transferred to glass tubes. Subsequently, the MIC, 2× MIC, and 4× MIC of two selected treatments (carvacrol and *O. compactum* EO) were added to the respective tubes. The tubes were placed in an incubator-shaker and agitated at a speed of 160 rpm for two hours. To collect the filtrate, the bacterial suspensions were passed through sterile 0.22 µm filters. The obtained filtrate was used to determine the concentration of nucleic acids and proteins by measurement of the absorbance at 260 and 280 nm wavelengths, respectively. A DeNovix spectrophotometer/fluorometer (DeNovix Inc., Wilmington, DE, USA) was used for these measurements. In addition, the bacterial suspension without any chemical treatments served as a negative control. The obtained values of nucleic acid and protein concentrations in the treated samples were compared with the negative control to assess the release of metabolites and evaluate the impact of the treatments on bacterial metabolism.

### 2.6. Determination of Phytotoxicity of Carvacrol to Olive Plants in Greenhouse

To assess the potential phytotoxic effect on olive plants (*Olea europaea* L.) we used the constituent phenol carvacrol, which was selected for its determined low toxic and strong antibacterial properties [32]. This experiment was conducted according to Obanor et al. [33], with a few modifications. A total of eight 2–3-year-old cv. Leccino plants per treatment were chosen for the experiment, which was set up in a greenhouse at the Institute of Agriculture and Tourism in Poreč, Croatia (45°13′17″ N; 13°36′9″ E), in June 2023, with irrigation included. To determine the dose-dependent phytotoxicity of carvacrol, two different concentrations were evaluated (MIC and 2× MIC). Water spraying was used as a control. The application of the treatment was performed using a garden hand sprayer, with a suspension volume of 1 L per treatment. The spraying process continued until the plants were fully covered with the suspension. The assessment of phytotoxic effects was focused on the occurrence of visible symptoms like necrotic lesions on the leaves. To determine the total damage to olive plants, we measured the percentage of damaged shoots showing symptomatic leaves (considered as the canopy) out of the total number of shoots developed on the olive plants. In addition, we determined the percentage of symptomatic leaves within the total number of leaves on the canopy. To determine if the fresh and dry biomass of leaves was affected, we took 20 symptomatic leaves per plant of each treatment, which is in total 160 leaves per treatment, and weighted the collected samples. The fresh weight of samples was determined immediately after collecting and dry weight was determined after drying the samples at 70 °C for 72 h.

### 2.7. Data Analysis

The data of the quantitative tests and the greenhouse experiment were analyzed by a one-way ANOVA model using the statistical software R (version 4.2.2). The analysis utilized the aov function to determine the statistical differences between treatments. The significance of differences was determined by a *p*-value threshold of ≤0.05. To control the false discovery rate, the *p*-values were adjusted using the Benjamini–Hochberg adjustment. Further comparisons of means were assessed with the contrast function from the “multcomp” package. The efficiency of the treatments was compared to the antibiotic and copper-based treatments by Dunnett’s test, using the DunnettTest function from the package “DescTools”. To create time–kill plots, the CFU/mL values were transformed to a logarithmic scale. All in vitro experiments were repeated at least three times, with three replications included.

## 3. Results

### 3.1. Qualitative Determination of Antibacterial Effects

The results presented in Table 1 indicate the different effects of antimicrobials at different concentrations, as determined by the disc diffusion susceptibility test (details in Table S1). The effectiveness of the treatments was assessed based on the size of the clearing zone (halo) around the disc. In summary, 34 out of 36 test treatments demonstrated either good or moderate antibacterial efficiency when compared to the effectiveness of standard antibiotics and copper-based preparations. The highest clearing zones, exceeding 20 mm, were observed for the undiluted forms of *T. vulgaris* and *O. compactum* EOs, and carvacrol, indicating their superior effectiveness compared to the copper-based treatment. While other treatments also proved effective, their efficiency was generally lower than that of the standard antibiotic and copper-based agents. It is worth noting that as the concentration decreased, the size of the clearing zones also decreased, indicating a concentration-dependent effect.

Conversely, the diluted form of *S. sclarea* essential oils and linalyl acetate exhibited even greater effectiveness compared to the undiluted form, and they were most potent at the lowest concentrations applied. We assume that this phenomenon may be influenced by improved diffusion through the solid growth medium when lower concentrations are applied to the discs. Furthermore, linalyl acetate belongs to the chemical group of esters, which differs in chemical structure from the other tested constituents. Additionally, the dilution in water might have affected the ionic strength and solubility of acetate, resulting in a decrease in salt concentration and thereby facilitating better diffusion through the agar medium in its diluted form [34].

**Table 1:** Inhibition of growth of bacterium *Pseudomonas savastanoi* pv. *savastanoi* (mm) using the disc diffusion method at three concentrations of selected antimicrobials (mean value ± standard error). Concentrations used were C1—pure/undiluted liquid of EOs or constituents or diluted solid form of the constituents; C2—diluted form of the EOs—20 mg/mL for *Thymus vulgaris* and *Origanum compactum* EOs, 40 mg/mL for other EOs, and 5 mg/mL for all constituents; C3—diluted form of the EO or EO constituent at a concentration of 5% (*v*/*v*).

Group	Antimicrobials	Inhibition of Growth of *Pseudomonas savastanoi* pv. *savastanoi* (Clearing Zone, mm)		
		C1	C2	C3
EOs	*Mentha* × *piperita*	8.56 ± 0.88 ^+^	9.01 ± 1.14 ^+^	8.45 ± 3.29 ^+^
	*Thymus vulgaris*	21.07 ± 2.96 ^++^	8.50 ± 1.04 ^+^	10.08 ± 0.98 ^+^
	*Origanum compactum*	20.29 ± 2.25 ^++^	8.90 ± 0.76 ^+^	10.18 ± 0.68 ^+^
	*Origanum majorana*	10.17 ± 1.13 ^+^	9.78 ± 0.64 ^+^	10.31 ± 0.56 ^+^
	*Salvia officinalis*	13.20 ± 2.52 ^+^	8.62 ± 0.77 ^+^	9.44 ± 0.76 ^+^
	*Salvia sclarea*	0.00 ± 0.00 ^x^	8.87 ± 0.96 ^+^	8.29 ± 0.82 ^+^
EOsC	DL-menthol *	9.63 ± 0.38 ^+^	8.30 ± 2.95 ^+^	9.30 ± 1.67 ^+^
	thymol *	9.05 ± 0.71 ^+^	10.52 ± 1.66 ^+^	10.01 ± 0.87 ^+^
	carvacrol	28.47 ± 3.99 ^++^	9.29 ± 0.70 ^+^	9.76 ± 0.83 ^+^
	linalyl acetate	0.00 ± 0.00 ^x^	8.54 ± 1.08 ^+^	7.79 ± 1.03 ^+^
	(-)-terpinen-4-ol	10.75 ± 2.16 ^+^	8.99 ± 0.90 ^+^	9.10 ± 0.89 ^+^
	α,β-thujone	11.73 ± 0.93 ^+^	10.22 ± 0.74 ^+^	9.20 ± 0.53 ^+^
Antibiotic	tetracycline	36.22 ± 0.00	n.t.	n.t.
Copper-based commercial pesticide	copper(I)oxide	16.32 ± 0.68	n.t.	n.t.
Negative control	sterile distilled water	0.00 ± 0.00	n.t.	n.t.

*—C1 for DL-menthol and thymol represents the diluted form of treatments (10 mg/mL) due to their crystalline structure; efficiency evaluated using adjusted scale of Aires et al. [35] ^x^—non-effective; +—clearing zone greater than zero but less than antibiotic and copper; ++—clearing zone higher than copper but less than antibiotic.

### 3.2. Quantitative Determination of Antibacterial Effect

**Figure 1:**
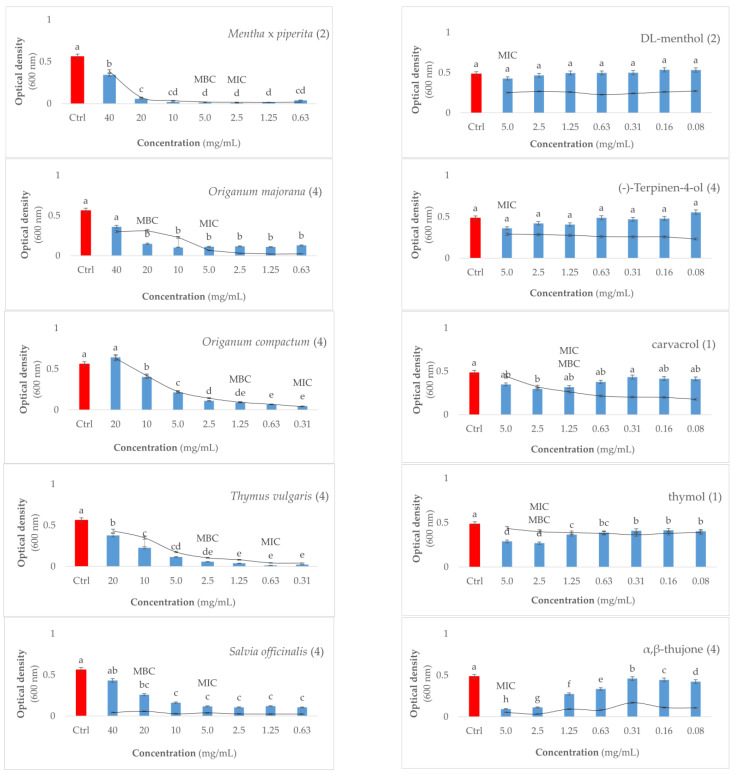

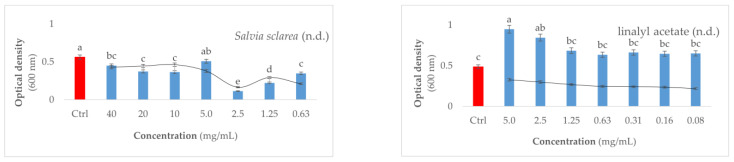
Determined minimal inhibitory (MIC) and bactericidal (MBC) concentrations of essential oils and their constituents against bacterium *Pseudomonas savastanoi* pv. *savastanoi*. OD_600_ values of antimicrobial treatments with bacterium are presented as blue bars, and OD_600_ of antimicrobial treatments without bacterium (controls) are presented as lines in the constructed plots. The red bars represent the negative control (bacterium without treatment). Numbers in brackets represent the value of MBC/MIC ratio of treatments; n.d.—not determined. Axis x represents the value of optical density (600 nm) and axis y shows the values of serially diluted concentrations used for the experiment expressed as mg/mL. Different letters above bars weight between concentrations and control (*Pss*) within each treatment at significance level of *p* < 0.05.

The results presented in Figure 1 indicate that all the tested antimicrobials have bactericidal rather than bacteriostatic properties, based on the calculated MBC and MIC ratio. The MIC values of EOs were generally lower compared to individual constituents, while the MBC was four times higher for most of used antibacterials (*T. vulgaris*, *O. compactum*, *O. majorana*, *S. officinalis*, (-)-terpinen-4-ol, and α, β-thujone). Interestingly, the EO constituents show a low MBC/MIC ratio for phenols like thymol, carvacrol, and DL-menthol. Exclusively, the isomers thymol and carvacrol exhibited bactericidal activity at MIC values of 2.50 and 1.25 mg/mL, respectively. We were not able to determine the MIC against *P. savastanoi* pv. *savastanoi* for *S. sclarea* EO and its constituent linalyl acetate, due to the observed visible growth of bacteria at the tested range of concentrations, which was confirmed after the inoculation of treatments on KB medium.

According to the one-way ANOVA analysis, the OD_600_ measurements showed significant differences between concentrations within each treatment. Specifically, the values decreased as the concentration of essential oils (EOs) decreased. In most cases, the measurements for the control treatments without bacteria were similar to those of treatments involving *Mentha* × *piperita*, *Origanum compactum*, and *Thymus vulgaris*. This suggests that at concentrations equal to or higher than the minimum inhibitory concentration (MIC), no bacterial growth occurred. However, there were exceptions to this pattern, particularly for *Salvia officinalis* and *Salvia sclarea* EOs, which led to the assumption that bacterial growth was not affected by these treatments. Nevertheless, the MIC of *S. officinalis* was successfully determined visually. As such, we assume that the deviations at higher concentrations of treatment might have been influenced by the mechanisms of action against the bacterial population, possibly affecting the density at higher concentrations and subsequently impacting the spectrophotometric readings when a small volume of 200 µL was transferred to the microtiter wells. Additionally, the MIC and minimum bactericidal concentration (MBC) values were significantly different from the control treatment (*Pss* and liquid medium) for all tested agents.

On the other hand, the EO constituents generally followed the expected pattern, with the OD_600_ decreasing as the concentration of the agents in the tubes increased. However, unlike the EOs, the MIC and/or MBC values did not statistically differ from the control *Pss* treatment, except in the cases of thymol and α,β-thujone treatments. It is important to note that although visible bacterial growth was not determined at the MIC concentration as shown in Figure 1, the lack of significant differences with the *Pss* control treatment resulted from the density of the treatment solutions.

**Figure 2:**
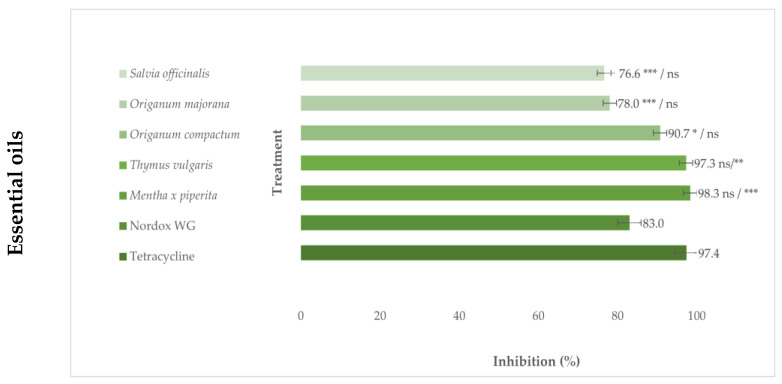

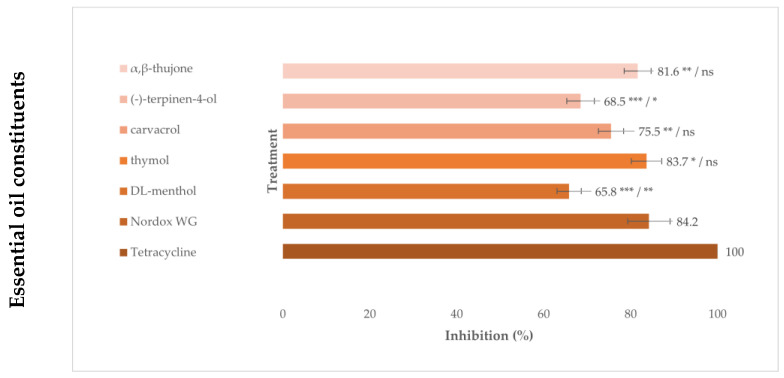
Percentage of *Pseudomonas savastanoi* pv. *savastanoi* growth inhibition in vitro at MIC concentrations of antimicrobials compared to standard antibiotic tetracycline and copper-based preparation Nordox WG, based on Dunnett’s comparison test with a significance level of * *p* ≤ 0.05, ** *p* ≤ 0.01, *** *p* ≤ 0.001, ns—not significant. Differences on the left and right side of the slash, as denoted on the bar of each treatment, represents statistical significance compared to antibiotic and copper treatment. Axis x represents the percentage of bacterial growth inhibition based on calculated OD_600_ values and axis y represents the treatments used in the experiment.

The results of the in vitro growth inhibition of *P. savastanoi* pv. *savastanoi* demonstrate that *T. vulgaris* and *M. piperita* EOs exhibited stronger antibacterial effect at their MIC, whilst the *S. officinalis, O. majorana* and *O. compactum* EOs were not significantly different compared to the commercial copper-based preparation (Nordox 75 WG) at the concentrations of 0.2% recommended by the manufacturer for application in olive orchards (Figure 2). Only *M. piperita* and *T. vulgaris* EOs did not significantly differ in their efficiency from the antibiotic treatment.

When considering individual constituents, all treatments were significantly weaker than antibiotic at their MIC values. The thymol, α,β-thujone, and carvacrol exhibited the same efficiency as the copper treatment. Further, the lowest inhibitory potential was determined for DL-menthol and (-)-terpinen-4-ol, which showed significantly lower effect compared to both the standard treatments. 

### 3.3. Time–Kill Assay

**Figure 3:**
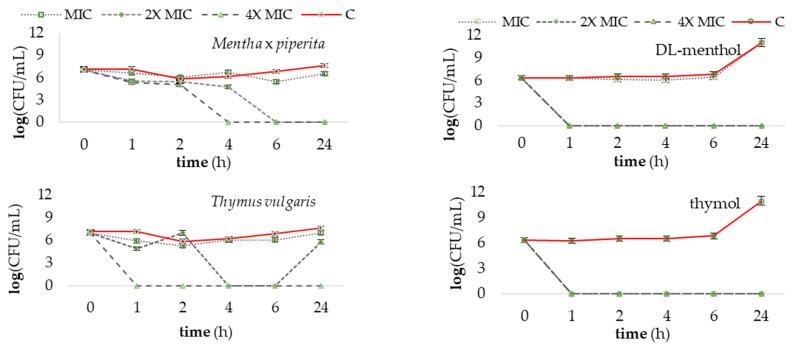

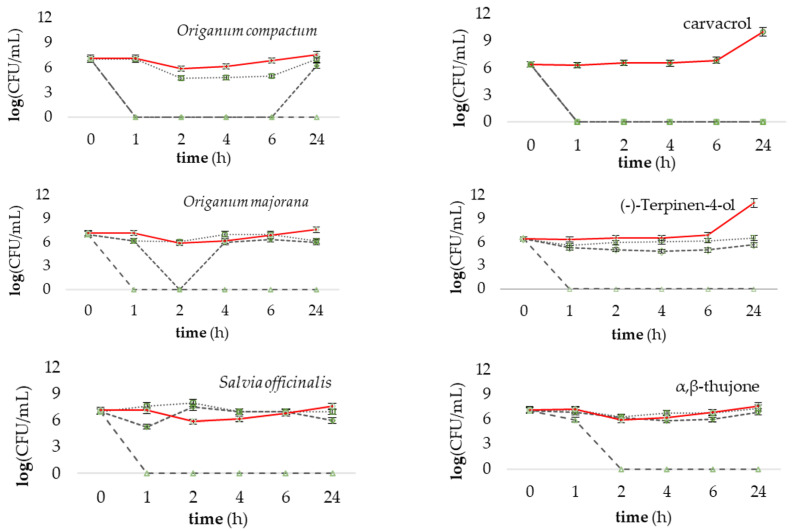
Survival of *Pseudomonas savastanoi* pv. *savastanoi* during exposure to EOs and its constituents in a 24 h time period (time–killing curves) at concentrations equal to MIC, 2× MIC, 4× MIC, and C (control without antimicrobials).

The results of the study (Figure 3) demonstrate a time- and concentration-dependent reduction in *P. savastanoi* pv. *savastanoi* bacterial population by ≥3log CFU/mL when exposed to increasing concentrations of the tested antibacterial agents. Among the treatments, EOs showed varied efficiency, with most treatments exhibiting initial antibacterial properties, except for *M. piperita* oil and α,β-thujone, which had a delayed response. However, at concentrations four times higher than the MIC, all treatments resulted in the complete inhibition of bacterial growth after one hour of incubation. Thymol and carvacrol were the most effective treatments, inhibiting bacterial growth at all tested concentrations compared to the non-treated control.

### 3.4. Leakage of Bacterial Cellular Metabolites

**Figure 4:**
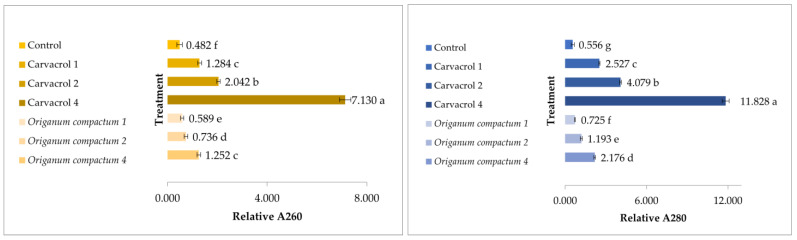
Assessment of nucleic acid and protein release after treatment with MIC, 2× MIC, and 4× MIC of *Origanum compactum* EO, its constituent carvacrol, and control (without antimicrobials) of bacterium *Pseudomonas savastanoi* pv. *savastanoi*. Relative A260 represents the nucleic acid release and relative A280 represents protein release. Treatments denoted by the same letter do not statistically differ according to one-way ANOVA analysis at *p* ≤ 0.05. Axis x represents the relative A260 and A280 values and axis y represents the treatments used in the experiment. Different letters along bars represent the statistical difference between concentrations and control (*Pss*) within each treatment at significance level of *p* < 0.05.

Figure 4 presents the relative absorbance values at 260 and 280 nm of the filtrate from *P. savastanoi* pv. *savastanoi* treated with different concentrations (MIC, 2× MIC, and 4× MIC) of *O. compactum* EO and its most concentrated constituent—carvacrol—along with the untreated bacterium. The relative absorbance values serve as an indicator of protein and DNA leakage from bacterial cells. Based on the obtained values, it is evident that carvacrol provoked a stronger leakage of proteins and DNA from bacterial cells compared to the EO during the experimental time period. Specifically, at the MIC concentration of *O. compactum* EO, there was no significant difference compared to the control treatment, indicating a limited protein and DNA release. On the other hand, carvacrol demonstrated better potential in terms of membrane rupture in the targeted bacterium. The EO exhibited the same level of nucleic acid release as the lowest tested concentration (MIC) of carvacrol only at the highest concentration of EO tested (4× MIC).

### 3.5. Phytotoxic Properties of Carvacrol in Planta

The mean weight values of fresh and dry leaves in control, carvacrol MIC, and carvacrol 2× MIC treatments were not statistically different (fresh leaf weight: 4.85, 4.35, and 4.62 g, respectively, and dry weight: 2.11, 1.76, and 1.91 g, respectively). According to the ANOVA, the variations in leaf weight were not statistically significant.

**Figure 5:**
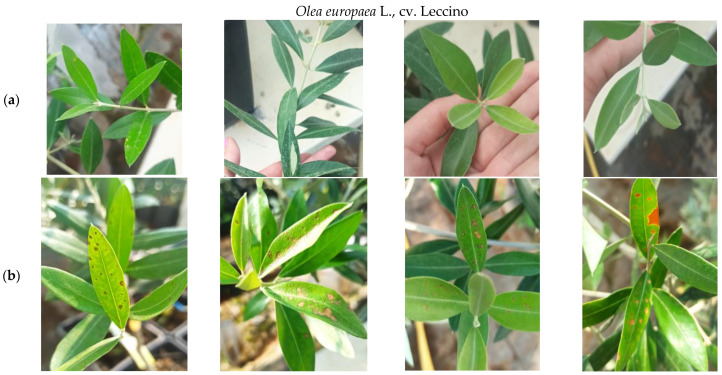
Observations of the phytotoxic effects of foliar application of different concentrations of carvacrol on olive plants cv. Leccino grown in greenhouse: (**a**) absence or poor damage on leaves of treated with 1.25 mg/mL of carvacrol; (**b**) damage of leaves on shoots of olive trees treated with 2.50 mg/mL of carvacrol (older leaves were undamaged or barely damaged).

The observations of olive plants in the greenhouse after the application of carvacrol, as presented in Figure 5, revealed that the symptoms of phytotoxicity manifested as necrotic lesions primarily on the leaves developing on the upper part of olive shoots, encompassing approximately one-third of the leaves per shoot. Older leaves, however, did not exhibit any visible damage, indicating that the phytotoxic effects were predominantly observed on the newly developing leaves. For the specific olive cultivar Leccino, the proportion of plants showing symptoms varied across different treatments. In the control treatment, no symptomatic plants were observed. In the MIC treatment, 75% of the olive plants exhibited symptoms of phytotoxicity. Finally, in the 2× MIC treatment, all olive plants displayed symptoms, resulting in a 100% proportion of symptomatic plants.

**Figure 6:**
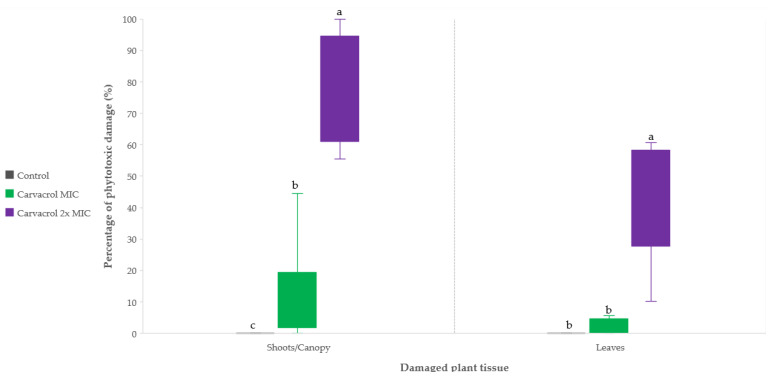
The percentage of phytotoxic damage observed on the shoots/canopy and leaves of young olive trees of cv. Leccino 14 days after the application of a carvacrol suspension at the minimum inhibitory concentration (MIC) and 2× MIC. Letters above boxplots denote statistically significant differences between treatments regarding olive canopy and leaf damage based on one-way ANOVA at significance level *p* ≤ 0.05.

Figure 6 shows the percentage of symptomatic canopy and the number of leaves showing symptoms as influenced by the sprayed concentration of carvacrol. The data show that the presence of symptoms, in the form of necrotic lesions on the adaxial surface of young leaves, increases with higher concentrations of carvacrol. After the application of the MIC of carvacrol, the majority of olive plants exhibited canopy damage ranging from 0 to 44.4% with 0–5.6% of the leaves being damaged per plant. However, after the application of 2× MIC of carvacrol, a larger proportion of the canopy (ranging from 55.6 to 100%) was affected, with 10.1–60.7% of the leaves being damaged per plant.

The mean percentage for canopy damage was determined to be 0.00, 13.4, and 77.3% for the control, MIC, and 2× MIC treatments of carvacrol, respectively. The analysis of variance indicated significant differences in canopy damage based on the treatment applied. The highest damage was observed in the 2× MIC carvacrol treatment compared to the control and MIC treatments, with a *p*-value of ≤0.001. Additionally, a significant difference was observed between the control and MIC carvacrol treatments at *p* ≤ 0.05.

The damage observed on the leaves follows a similar pattern, with a significantly higher percentage of symptoms observed in the 2× MIC carvacrol treatment compared to the MIC and control treatments. However, the application of MIC carvacrol did not show a statistically significant difference compared to the untreated control.

## 4. Discussion

The presented study evaluated the antimicrobial effect of twelve bioactive compounds selected for testing against *Pseudomonas savastanoi* pv. *savastanoi*, a bacterial pathogen that negatively impacts olive cultivation and leads to a reduction in yield and quality [36]. Out of the twelve compounds tested in vitro, ten had an effect on the pathogen, hinting their potential for controlling this economically significant disease in planta in olive-growing regions. Regarding the *Mentha* × *piperita* EO, it exhibited good antibacterial activity, along with its constituent menthol, which demonstrated strong antibacterial properties and exhibited bactericidal activity within one hour of exposure to concentrations of 10 and 20 mg/mL, respectively. This finding aligns with previous reports suggesting that the *Mentha* species, although generally less effective compared with *Thymus* or *Origanum* genera, can still exhibit efficacy depending on the concentration of menthol as the predominant constituent in their EOs [7,9,10]. In addition, *Mentha* EO exhibits unique synergistic features, which can enhance the effectiveness of other EOs and antibiotics when used in combination [7]. Further research is needed to explore the potential of reducing commercial pesticide dosages through the use of *Mentha* EO in mixtures. Similarly, species from the *Salvia* genus, which are abundant within the Lamiaceae family, generally exhibit lower efficacy against Gram-negative bacteria compared to Gram-positive bacteria [7,16,37].

However, this study underscores the significance of identifying specific *Salvia* species with potential biopesticidal properties, indicating that further evaluation and development of biopesticides derived from *Salvia* species may be worth exploring. Specifically, our observations reveal that *S. officinalis*, particularly the chemotype α,β-thujone, exhibited higher efficacy (demonstrating bactericidal effects at 40 mg/mL) than the chemotype linalyl acetate found in *S. sclarea*. It is important to note that while the diluted *S. sclarea* EO and constituent linalyl acetate (as shown in Table 1) appeared efficient in the diluted form, some studies have suggested its ineffectiveness against bacterial species within the *Pseudomonas* genus [38]. Furthermore, linalyl acetate displayed interesting results in the broth dilution method, with significantly higher OD_600_ values at all tested concentrations, implying a potential stimulation of bacterial growth. This phenomenon warrants further investigation into the mechanism of action of this constituent in future research. Nevertheless, the lower susceptibility of certain plant-derived treatments may be linked to the genetic profile of *Pss*, as Italian strains are considered more virulent than strains infecting olives in other Mediterranean olive-growing regions [39]. Another factor influencing the determination of antibacterial effect is the methodology employed. Limitations in testing plant derivates arise from the non-polar nature of most agents, which can hinder their diffusion through agar medium and lead to possible interactions with ingredients present in the growth medium [40]. To our knowledge, there is no comprehensive research on the antibacterial effect of plant derivates against *Pss* addressing this interaction. Nevertheless, the disc diffusion method is frequently used to assess the susceptibility of numerous bacterial species to plant derivates, resulting in variations in testing outcomes in the same and different laboratories [24,40,41,42]. This emphasizes the need to optimize this method to obtain more uniform results across laboratories. In addition, considering the optimization of the methodology, the hydrophobic nature of EOs and some of their constituents may play a significant role in antibacterial testing. Specifically, when testing larger non-polar molecules in diluted forms, higher temperatures of incubation may be required to increase their solubility compared to smaller molecules [34]. This aspect should be explored in future research to enhance the standardization of protocols for testing the antibacterial effects of plant-derived agents such as EOs and their constituents.

On the other hand, findings obtained in the current study confirm that carvacrol and thymol, both as individual constituents and in the carvacrol-thymol chemotypes of *O. compactum* and *T. vulgaris*, exhibit the most potent antibacterial effect [7,11,37,43,44,45]. As such, we observed that *O. compactum* and *T. vulgaris* EOs achieved a 99.9% reduction in bacterial growth when applied at 2× MIC concentrations within a 6 h timeframe (Figure 3). However, it is worth noting that bacterial regrowth was observed after this specified period, indicating that the bioactivity of EOs is dependent on both concentration and time of bacterial exposure to them. This phenomenon can be attributed to the volatile nature of EOs and the potential antagonistic interactions among diverse constituents in their chemical profile, which can influence their overall effectiveness. The results of the leakage of cellular metabolites for the *O. compactum* EO and its concentrated phenol carvacrol, presented in Figure 4, support this observation due to the observed higher effect in cell membrane disruption of carvacrol compared with *O. compactum* EO. In a study by Bozkurt et al. [13], EOs rich in carvacrol and thymol also demonstrated the highest potential against *Pss*. Interestingly, the study also highlighted the potential of the terpinen-4-ol chemotype *O. majorana* EO, which exhibited greater inhibition of bacterial growth compared to our findings. However, it is important to consider the use of different concentrations in between the two studies. The concentration of terpinene-4-ol used in the mentioned study was higher in the *O. majorana* EO (31.67%) compared to our study (22.54%). This suggests that the antibacterial activity of terpinen-4-ol is indeed concentration-dependent, as supported by our results showing that a concentration of 4× MIC of the chemical constituent and *O. majorana* EO exhibited bactericidal properties against *Pss*. Moreover, the volatile nature of EOs may impede their ability to penetrate and damage bacterial cells effectively, thus poorly affecting bacterial growth or allowing for regrowth over time. To address these limitations and enhance the effectiveness and prolonged antibacterial activity of EOs, their encapsulation in chitosan nanoparticles has been proposed as a potential solution [45,46,47]. For example, a study by Baldassarre et al. [47] determined that thymol loses its inhibitory potential in vitro over time against *X. fastidiosa* subsp. *pauca*, specifically when used alone at concentrations of 0.125–1.000 mg/mL; however, it resembles significantly efficient effects in the encapsulated form. This observation may provide insight into the regrowth of *Pss* observed at the 2× MIC concentration of *T. vulgaris* EO after a 6 h exposure to the treatment in our study. It suggests that the activity of thymol chemotype EOs may diminish over time, possibly explaining the regrowth of the bacteria despite the initial antibacterial effects observed.

Recent studies have elucidated the mechanism of action of phenolic compounds, such as carvacrol and thymol, as disruptors of bacterial cell membranes. These constituents have been found to cause significant leakage of metabolites and subsequent bacterial death in in vitro studies [7]. The results of our study targeting the bacterium *Pss* support this mode of action. Nevertheless, carvacrol and thymol exhibited potent antibacterial activity across all tested concentrations, resulting in a reduction of over 3log after just one hour of exposure, which was consistent during the investigated time of exposure CFU/mL. Notably, the antibacterial efficacy of carvacrol and thymol was not concentration-dependent in our case, indicating their strong activity even at extremely low concentrations (1.25 and 2.5 mg/mL, respectively). The antimicrobial effects of EO constituents, such as carvacrol, can be attributed to their lipophilic and hydrophobic properties, which enable them to penetrate the cell membranes of microorganisms. Once inside the cells, they can exert various antimicrobial actions, including cell lysis, inhibition of protein synthesis, cytoplasmic coagulation, increased membrane permeability, and alterations in cell pH gradient [21,48]. This suggests that carvacrol specifically, among the constituents present in the EOs, plays a major role in the observed antibacterial activity.

Although EOs and their constituents have demonstrated equal or significantly higher antibacterial activity compared to copper-based treatment at their MIC values, there is still a lack of understanding of their potential phytotoxic effects on woody plants, particularly in the case of olive trees. To fill this gap in the knowledge, we conducted preliminary evaluations to investigate the potential phytotoxic properties of carvacrol, a prominent antibacterial constituent, hereby chosen for its wide use in disciplines other than agriculture and its position as one of the most studied EO constituent from an antibacterial perspective, as well [49,50]. Additionally, carvacrol can be converted into less toxic carvacryl acetate via acetylation [50]. However, to our knowledge the antibacterial effect of carvacryl acetate has not been studied against plant pathogenic bacterial species. It is important to note that the evaluation of phytotoxicity is a critical aspect when considering the practical application of any antimicrobial agent in plant production. Overall, our preliminary evaluations shed light on the potential use of this constituent in the management of olive diseases. Despite the expected phytotoxic effects on olive plants, as reported in studies focusing on weed management [8], we aimed to evaluate the potential phytotoxic properties of carvacrol treatment specifically on olive trees. Terpenoids, including terpenic phenols like carvacrol and thymol, have been found to induce alterations in plants, similar to the effects of salinity stress. These alterations include increased abscisic acid content, stomatal closure, heat accumulation at the leaf lamina, mitosis inhibition, removal of the cuticular wax layer, and microtubule polymerization [51]. Consistent with this mode of action, our study revealed the occurrence of necrotic lesions on the young leaves of developing shoots of olive trees (cv. Leccino) after just one day of carvacrol treatment. The occurrence of phytotoxicity was found to be dose-dependent, with a 100% occurrence observed when the treatment was applied at a concentration two times higher than the MIC, while the control treatment with water did not exhibit any symptoms. Furthermore, we quantified the percentage of symptomatic shoots and leaves for the 2× MIC (2.50 mg/mL) and MIC (1.25 mg/mL) treatments, which were determined to be 12.9% and 2.8% for shoots, and 78.3% and 34.3% for leaves, respectively. These results emphasize that carvacrol can be toxic to olive plants, focusing on the importance of evaluation of appropriate concentrations and application methods to effectively control olive knot disease without causing significant damage to olive canopies, and while efficiently suppressing bacterial growth on hosts. Based on these preliminary findings further investigations are warranted. Notably, future experiments on olives could be conducted in early spring or in the dormant phase of vegetation to determine the potential effect of temperature or plant phenological phase as causative variables in phytotoxic damage by carvacrol. Other studies could include an evaluation of treatment efficacy prior to artificial infection of olive plants under controlled conditions using various concentrations, adjuvants, or formulations. Additionally, experiments considering water stress, different temperature and humidity regimes, and field conditions should be conducted to provide a comprehensive understanding of treatment efficiency. By conducting such studies, we can contribute to the development of effective and sustainable strategies for managing olive knot disease in the near future. It is essential to strike a balance between controlling the disease and minimizing phytotoxic effects, preserving the health and productivity of olive trees, but also other fruit trees and agricultural crops.

## 5. Conclusions

The findings of this study highlight the potential use of bioactive compounds in managing the economically significant bacterium *Pseudomonas savastanoi* pv. *savastanoi* (*Pss*) which causes the olive knot disease. Among the twelve compounds tested, ten showed promising antibacterial effects, suggesting their potential as biopesticides. Notably, the *M. piperita* EO exhibited good bactericidal properties through its constituent menthol, demonstrating the importance of specific constituents present in EOs. Nevertheless, phenols carvacrol and thymol, both as individual constituents and in specific chemotypes of *O*. *compactum* and *T*. *vulgaris* EOs, were observed to be potent antibacterials. However, their effectiveness was influenced by concentration and time of exposure, possibly due to the volatile nature of EOs and their complex chemical profiles. This study also highlights the potential of certain *Salvia* species, such as *S*. *officinalis*, as biopesticides, emphasizing the need for further research on them. Preliminary evaluations of the phytotoxicity of carvacrol indicated that this agent can be toxic to olive trees at concentrations higher than MIC (1.25 mg/mL), highlighting the need for further investigations under various conditions and concentrations to develop effective and sustainable formulations for managing olive knot disease and minimizing damage to the olive canopy. Genetic variability within *Pss* strains, mechanism of action, chemical group of constituents and methodological approach, were acknowledged as factors that may influence the antibacterial effect of plant-derived agents, emphasizing the need for further optimization.

## Data Availability

The data presented in this study are available on request from the corresponding author.

## Supplementary Materials

The following supporting information can be downloaded at: https://www.mdpi.com/article/10.3390/microorganisms11112735/s1, Table S1: The list of antimicrobials selected for antibacterial testing against *Pseudomonas savastanoi* pv*. savastanoi* in concentrations specified; Table S2: Chemical profile of commercial essential oils used as antimicrobials.
