# Supplementary material for: The Antibacterial Effect of Selected Essential Oils and Their Bioactive Constituents on Pseudomonas savastanoi pv. savastanoi: Phytotoxic Properties and Potential for Future Olive Disease Control
Source: Microorganisms. 2023 Nov 9;11(11):2735. doi: 10.3390/microorganisms11112735 (PMC10673089; doi:10.3390/microorganisms11112735)
Supplement: Supplementary file 1 [file microorganisms-11-02735-s001.zip › microorganisms-2674093-supplementary.pdf]

microorganisms MDPI

Article

The Antibacterial Effect of Selected Essential Oils and Their Bioactive Constituents on *Pseudomonas savastanoi* pv. *savastanoi*: Phytotoxic Properties and Potential for Future Olive Disease Control

Laura Košćak [1], Janja Lamovšek [2], Edyta Đermić [3] and Sara Godena [1,*]

[1] Laboratory for plant protection, Department of Agriculture and Nutrition, Institute of Agriculture and Tourism, Carlo Hugues 8, 52440 Poreč, Croatia
[2] Agricultural Institute of Slovenia, Hacquetova ulica 17, 1000 Ljubljana, Slovenia
[3] Department of Plant Pathology, Division of Phytomedicine, Faculty of Agriculture, University of Zagreb, Svetošimunska cesta 25, 10000 Zagreb, Croatia
* Correspondence: sara@iptpo.hr

Supplementary Materials

Table S1. The list of antimicrobials selected for antibacterial testing against *Pseudomonas savastanoi* pv. *savastanoi* in concentrations specified.

Group	Antimicrobials	Concentration (mg/ mL)		
		C1	C2	C3
Essential oils (EOs)	*Mentha* x *piperita L.*	Undiluted	40	50
	Thymus vulgaris L.	Undiluted	20	50
	Origanum compactum L.	Undiluted	20	50
	Origanum majorana L.	Undiluted	40	50
	Salvia officinalis L.	Undiluted	40	50
	Salvia sclarea L.	Undiluted	40	50
Essential oil constituents (EO constituent)	DL-menthol*	10	5.0	50
	thymol*	10	5.0	50
	carvacrol	Undiluted	5.0	50
	linalyl acetate	Undiluted	5.0	50
	(-)-Terpinen-4-ol	Undiluted	5.0	50
	α,β - thujone	Undiluted	5.0	50
Antibiotic	tetracycline	0.5	-	-
Copper-based commercial pesticide	copper(I)oxide	2.0	-	-
Control	Sterile distilled water	-	-	-

*10mg/mL – chemical diluted due to solid crystalline structure.

Table S2. Chemical profile of commercial essential oils used as antimicrobials. The chromatographic profile of *Mentha* x *piperita*, *Thymus vulgaris*, *Origanum compactum*, *Origanum majorana* and *Salvia sclarea* were obtained from PRANARŌM international (Belgium), and *Salvia officinalis* from Fagron (Croatia).

	**Concentration** (% peak area)					
**EO constituents**	***Mentha* x *piperita***	***Thymus vulgaris***	***Origanum compactum***	***Origanum majorana***	***Salvia officinalis***	***Salvia sclarea***
α-pinene	0.91	1.16	0.56	0.72	-	0.11
β-pinene	1.08	0.25	0.12	-	-	0.13
Sabinene	0.46	0.01	-	5.26	-	0.03
Limonene	2.47	0.37	0.22	1.76	-	0.62
1,8-cineole	5.51	0.05	0.09	0.19	12.9	0.02
menthone	24.08	-	-	-	-	-
menthofurane	2.07	-	-	-	-	-
isomenthone	3.64	-	-	-	-	-
menthyl acetate	5.48	-	-	-	-	-
neomenthol	2.91	-	-	-	-	-
β-caryophyllene	3.05	2.09	1.72	-	-	1.46
neoisomenthol	0.61	-	-	-	-	-
menthol	36.37	-	-	-	-	-
pulegone	1.21	-	-	-	-	-
α-terpineol	0.55	0.31	0.20	2.90	-	2.00
Germacrene D	1.08	-	-	-	-	3.34
piperitone	1.04	-	-	-	-	-
α-thuyene	0.06	1.22	0.90	0.70	-	-
camphene	0.03	1.15	0.09	0.04	-	0.02
β-myrcene	0.23	1.52	1.60	1.80	-	0.84
α-terpinene	0.18	1.23	1.64	9.50	-	0.01
γ-terpinene	0.35	10.05	14.12	15.14	-	0.02
p-cymene	0.18	17.03	7.84	1.45	-	0.02
camphre	-	0.66	0.09	-	19.0	-
linalool	0.17	4.52	1.38	1.87	-	-
linalol	-	-	-	-	-	18.19
Terpinene-4-ol	0.64	1.55	0.42	22.54	-	0.02
borneol	-	2.07	0.17	-	2.8	0.05
thymol	0.03	45.73	8.21	0.02	-	-
carvacrol	-	4.10	57.58	0.04	-	-
β-phellandrene	-	0.23	0.19	1.88	-	-
terpinolene	0.10	0.12	0.08	3.42	-	0.14
Trans-thuyanol	0.24	0.20	0.19	5.32	-	-
E-solanone	-	-	-	1.64	-	-
Cis-thuyanol	0.04	0.10	-	9.01	-	-
linalyl acetate	-	-	-	3.75	-	62.94
Trans-*p*-menth-2-en-1-ol	0.03	0.03	0.02	1.02	-	-
β-caryophyllene	-	-	-	2.87	-	1.46
Cis-*p*-menth-2-en-1-ol	-	0.03	0.02	0.82	-	-
bicyclogermacrene	-	-	-	1.50	-	0.47
Cis-piperitol	-	-	-	0.55	-	-
α-, and β-thuyone	-	-	-	-	33.8	-
bornyl acetate	-	0.07	-	0.06	1.9	0.50
